# Synthesis and Characterization of Constrained Geometry Oxygen and Sulphur Functionalized Cyclopentadienylchromium Complexes and Their Use in Catalysis for Olefin Polymerization

**DOI:** 10.3390/molecules22050856

**Published:** 2017-05-22

**Authors:** Ruiguo Zhao, Jun Ma, Hao Zhang, Jiling Huang

**Affiliations:** 1 School of Chemistry and Chemical Engineering, Inner Mongolia University, Hohhot 010021, China; zhaoruig@imu.edu.cn; 2 The Laboratory of Organometallic Chemistry, East China University of Science and Technology, Shanghai 200237, China; majun197906@sohu.com

**Keywords:** CGC-organochromium complexes, oxygen functionalized cyclopentadienyl, olefin polymerization

## Abstract

A series of constrained geometry O-functionalized cyclopentadienylchromium complexes (**1**–**6**) and a S-functionalized cyclopentadienylchromium complex (**7**) were first synthesized, characterized, and tested as catalyst precursors for the olefin polymerization. In the presence of MAO, the complexes exhibited high catalytic activity for the polymerization of ethylene. It is shown that ligand variations can have a substantial effect on catalyst activity and stability. The effect of Al/Cr ratio on catalytic activity was also studied.

## 1. Introduction

**Figure 1:**
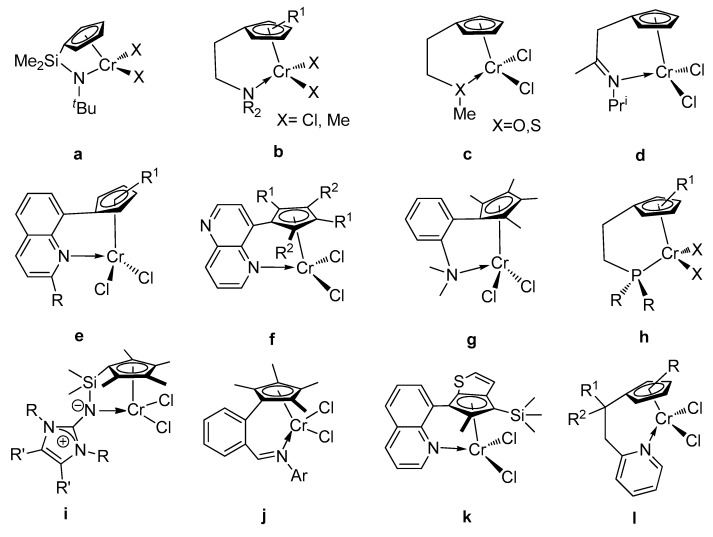
Constrained geometry Cyclopentadienylchromium Complexes.

Olefin polymerization by homogeneous transition metal complex catalysts attracts particular attention in the field of organometallic chemistry, catalysis, and polymer chemistry. Many reports have focused on the use of various transition metal complexes [1,2,3,4,5]. Chromium catalysts played a key role in the early development of heterogeneous catalysts for the polymerization of alkenes. It has been well known that the chromium-based heterogeneous catalysts, such as Phillips catalysts (Cr_2_O_3_/SiO_2_) [6] and the Union Carbide Unipol catalysts (Cp_2_Cr /SiO_2_) [7,8], have been used in industrial production of high-density polyethylene (HDPE) since the 1950s. Contrary to the Ziegler-Natta system, few studies have reported on the mechanism and nature of the active species in these chromium catalytic systems. It is mainly because the chemistry of Cr(III) is particularly difficult to study as a result of its paramagnetic nature. In recent years, some of the most significant advances in Cp-based chromium catalyst systems have been made using precatalysts that bear an additional neutral donor and bridged the Cp unit [9,10,11,12,13,14,15,16,17,18,19,20,21,22,23,24,25], so-called CGC system (CGC = Constrained Geometry Catalyst). They have already been used as efficient catalyst precursors for ethylene or 1-hexene homo (co)-polymerization, which are listed in Figure 1. However, only a couple of reports concerning O-functionalized Cp-based chromium catalyst have been published [10].

**Figure 2:**
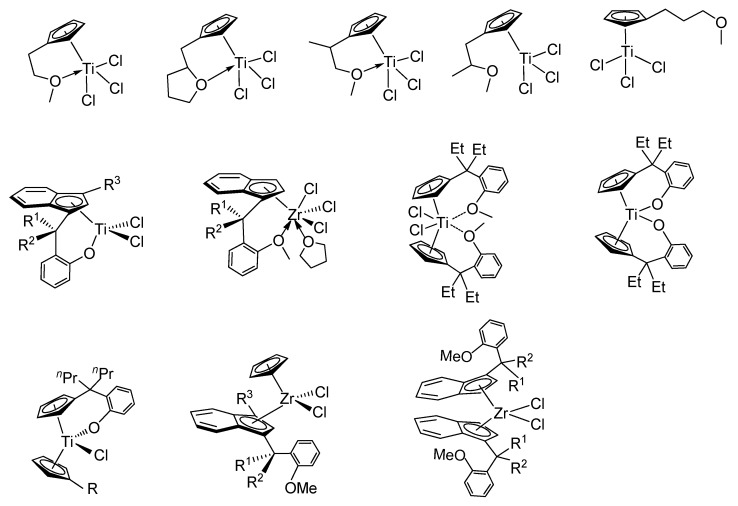
Ti, Zr complexes were reported by our group.

Since 1985, our group has been devoted to the research of O-containing Ti, Zr complexes. In our previous work, we introduced methoxyl ethyl, tetrahydrofurfuryl, and *ortho*-methoxyl benzyl type groups to the Cp (Ind) ligand, and found they showed a good tendency to form a chelate structure under moderate conditions in the synthesis of corresponding Ti, Zr complexes (Figure 2) [3,26,27,28,29].

With regard to the relevance to the above Cp-based chromium catalyst and our primary interest [25], we are interested in exploring the possibility of O-functionalized and S-functionalized cyclopentadienylchromium complexes as the olefin polymerization catalyst. Here, we describe the synthesis of a range of O-functionalized and S-functionalized cyclopentadienylchromium complexes catalysts and evaluate the effect of variations in the ligand system on the performance in ethylene polymerization catalysis.

In this paper, we wish to discuss the synthesis, structure and olefin polymerization details of novel *ortho*-methoxyl benzyl, *furyl*, and the *thiofuryl* substituted cyclopentadienylchromium complex. As a result of these studies, a proposal for the pathways of generation and action of the species responsible for the ethene polymerization could be formulated, and catalysts have been obtained that can produce >100 kg of ethene polymerization product/(mol of Cr) under moderate conditions.

## 2. Results

### 2.1. Synthesis and Characterization of Chromium Complexes

**Scheme 1:**
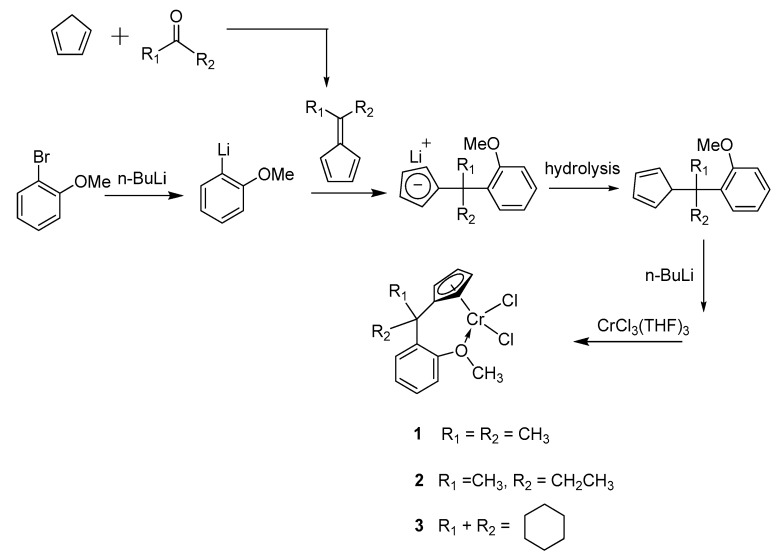
Route of the synthesis of cyclopentadienylchromium complexes **1**–**3**.

The route of the synthesis of the *ortho*-methoxyl benzyl substituted cyclopentadienyl chromium complex **1**–**3** is summarized in Scheme 1. The *ortho*-methoxyl benzyl substituted cyclopentadienyl ligand is readily obtainable from the reaction of 6,6-dialkylfulvene with the appropriate *ortho*-methoxyl benzyl lithium salts [27]. The reaction of these ligands with CrCl_3_ (THF)_3_ leads to the half-sandwich chromium complexes **1**–**3**, which were isolated in moderate yield. The IR spectra of these complexes also prove that the oxygen atom is coordinated to the chromium atom (see Supplementary Materials).

**Scheme 2:**
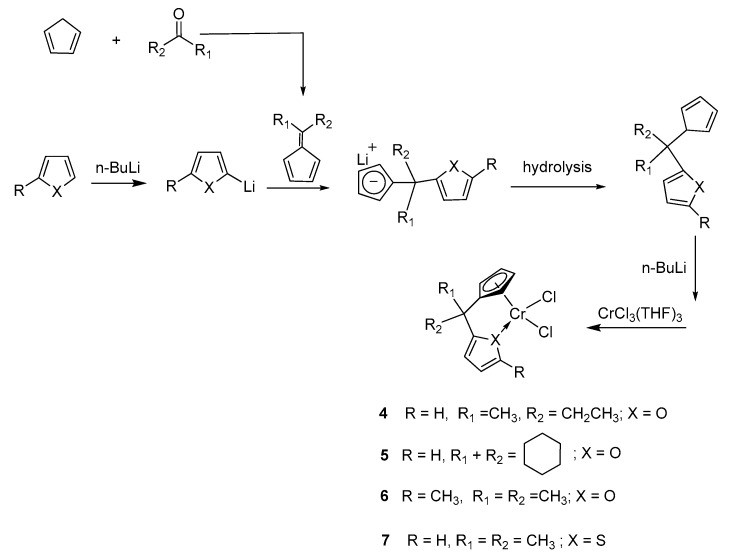
Route of the synthesis of cyclopentadienylchromium complexes **4**–**7**.

The routes employed for the synthesis of the *furyl* and the *thiofuryl* substituted cyclopentadienylchromium complexes are summarized in Scheme 2. These ligands were readily obtainable from the reaction of 6,6-dialkylfulvene with the appropriate *furyl* or *thiofuryl* lithium salts [27,29].

### 2.2. Polymerization of Olefin Use ***1**–**7***

The ethylene polymerization results using complexes **1**, **2**, **3**, **4**, **5**, **6**, and **7** as catalyst precursor in the presence of MAO are shown in Table 1. The catalytic activities of complexes **1**, **2**, **4**, and **6** were similar to those of Cp_2_TiCl_2_ e.g., 10^5^ gPE/mol M·h. In addition, the catalytic activities of complex **7** were similar to those of Cp_2_ZrCl_2_, e.g., 10^6^ gPE/mol M·h. Among them, complex **7** showed the highest activity as 1.96 × 10^6^ gPE/mol Cr·h for ethylene polymerization. The ligand structure and the molar ratio of Al/Cr influence both the activities of the complexes and the molecular weights of resultant polyethylenes.

The data in Table 1 demonstrate that the steric and electronic effects of the substituents on side chain of cyclopentadienyl the ligand exert significant influence on the catalytic activities of these complexes. Complexes **1**, **2** and **4**, **6** with *ortho*-methoxyl benzyl and *furyl* on side chain of the cyclopentadienyl show much lower activities than complex **7** with *thiofuryl* substituents for ethylene polymerization under same conditions (entries 3, 4, 13, 14 versus entries 15, 16), suggesting that the electronic effect of the substituents may have played a significant role in ethylene polymerization. This may be due to the coordination atom from O to S which makes the coordination capacity of coordination atom increase and the corresponding complex catalytic activity increases as a result. At the same time, with the increase of the steric hindrance on the side chain carbon bridge of complexes **1** and **2**, an obvious increase of the catalytic activity is observed when double *Me* groups are replaced by *Me* and *Et* groups. The increase in the steric hindrance on the carbon bridge may be more favorable for the coordination of the suspended group with the metal center [29]. However, the space effect of side chain of the cyclopentadienyl ring is inverted for *cyclohexyl* substituted complexes **3** and **5**. We speculate that this may be because the role of carbon bridge steric hindrance is too large so that the coordination space of the catalytic activity center is too congested and it is difficult for ethylene to insert, which results in a decrease of the catalytic activity.

It is found that the molecular weights of the resultant polymers are closely dependent on the structure of the catalysts. In general, the increase of the steric hindrance of the substituents in the ligand framework results in a decrease of the molecular weights of the obtained polymers. For example, under the polymerization conditions of Al/Ti = 1000 and 2000, with the variation of the substituent of side chain the cyclopentadienyl ring from double *Me* (**1**) to *Me* and *Et* (**2**), a decrease of *M*_η_ values of the resulted polyethylenes from 8.96~5.62 × 10^4^ g/mol to 7.73~4.40 × 10^4^ g/mol could be observed (entries 3, 4, 5, 6, respectively). GPC measurement of a polymer sample obtained with complex **7** indicated *M*_n_ = 4.92 × 104 g/mol with *M*_w_/*M*_n_ = 1.89 (entry 16 in Table 1). Moreover, the obtained PEs possesses melting points in the range 133–135 °C, typical for linear polyethylene. [30,31]

As observed for most of the catalytic olefin polymerization systems, the activity of these chromium complexes toward ethylene polymerization is also influenced by the molar ratio of Al/Cr. With the increase of Al/Cr ratio from 1000 to 2000, the activity of complex **7** is increased from 1.10 × 10^6^ g PE/mol Cr·h to 1.96 × 10^6^ g PE/mol Cr·h (entries 15 and 16). A similar trend is also found for the other complexes. The influence of the Al/Cr molar ratio on the polymer molecular weights was also investigated. The results shown in Table 1 indicate that, in general, under the same conditions, the molecular weight of the obtained PE decreases with the increase of the Al/Cr molar ratio. From the ^1^H-NMR spectrum of a typical polyethylene sample obtained with **7**, (see Supplementary Materials) no obvious alkenyl hydrogen signals could be detected, implying that the dominant termination process is chain transfer to aluminum instead of the β-hydride elimination reaction [31,32].

## 3. Materials and Methods 

### 3.1. General Experimental Procedures

All operations were carried out under an argon atmosphere using the standard Schlenk techniques. Tetrahydrofuran (THF), diethyl ether, toluene, and n-hexane were freshly distilled from sodium benzophenone ketyl under argon prior to use. Methylene chloride was distilled over P_2_O_5_ and CaH_2_ under nitrogen. Methylaluminoxane (MAO) was purchased from Wittco (Baltimore, MD, USA).

All complexes were characterized by FT-IR, EA, and MS. ^1^H-NMR spectrum of typical polymer sample was recorded on a GRMINI-400 NMR spectrometer (Varian, Palo Alto, CA, USA) at 100 °C using a mixture solvent of *o*-dichlorobenzene and C_6_D_6_ (4:1). IR spectra were recorded on a Nicolet MAGNA-IR 550 spectrometer (Thermo Fisher Scientific, Waltham, MA, USA) as KBr pellets. Mass spectra were obtained at 70 eV using a HP 5989A mass spectrometer. Elemental analyses were performed on an EA-1106 spectrometer.

### 3.2. Synthesis of Complexes ***1**–**7***

Synthesis of complex **1**: Ligand **1a** was prepared according to the literature [27]. To a solution of 2.13 g (10.0 mmol) ligand **1a** in 20 mL THF in the flask, a solution of butyl lithium 8.19 mL (1.22 mol/L, 10.0 mmol) in THF was dropwise added at −78 °C. After complete addition the reaction mixture was stirred for 12 h. Then the resulting solution was added to the suspension of CrCl_3_ (THF)_3_ 3.71 g (10.0 mmol) in 45 mL of THF at −78 °C. The mixture was stirred overnight. The color of the reaction mixture changed from purple to blue then to dark green. After the solvent was removed under vacuum, toluene was added to precipitate LiCl, and the organic layer was concentrated to 30 mL. At low temperature, 0.46 g dark green crystals were obtained in 13.6% yield. EI-MS (m/e): 335 (75, M), 300 (100, M-Cl), 264 (52, M-2Cl), 235 (5, M-2CH_3_-2Cl), 213 (12, M-2Cl-Cr), 198 (11, M-CH_3_-2Cl-Cr), 183 (13, M-2CH_3_-HCl-Cr), 108 (47, PhOCH_3_). IR(cm^−1^, KBr): 3109 m, 3092 m, 3070 w, 2995 m, 2960 m, 2875 w, 1482 s, 1447 s, 1405 w, 1363 m, 1287 m, 1241 m, 1206 m, 1155 m, 1136 m, 1080 m, 1047 w, 1032 m, 973 s, 933 w, 839 s, 798 s, 761 s, 694 m. Calc. for C_15_H_17_Cl_2_CrO: C, 53.59%; H, 5.10%. Found: C, 53.11%; H, 5.16%. HRMS Calc. for C_15_H_17_Cl_2_CrO = 335.0063. Found: C_15_H_17_Cl_2_CrO = 335.0062.

Synthesis of complex **2**: Ligand **2a** was prepared according to the literature [27]. To a solution of 2.27 g (10.0 mmol) ligand **2a** in 20 mL THF in the flask, a solution of butyl lithium 8.19 mL (1.22 mol/L, 10.0 mmol) in THF was dropwise added at −78 °C. After complete addition the reaction mixture was stirred for 12 h. Then the resulting solution was added to the suspension of CrCl_3_ (THF)_3_ 3.56 g (9.58 mmol) in 45 mL of THF at −78 °C. The mixture was stirred overnight. The color of the reaction mixture changed from purple to blue then to dark green. After the solvent was removed under vacuum, toluene was added to precipitate LiCl, and the organic layer was concentrated to 30 mL. At low temperature, 1.18 g dark green crystals were obtained in 35.0% yield. EI-MS (m/e): 349 (100, M), 314 (59, M-Cl) 279 (15, M-2Cl), 250 (6, M-2Cl-Et), 227 (10, M-2Cl-Cr), 198 (11, M-CH_3_-2Cl-Cr), 183 (13, M-2CH_3_-2Cl-Cr), 108 (4, PhOCH_3_). IR (cm^−1^, KBr): 3112 w, 3097 s, 3043 w, 2973 s, 2937 w, 2878 w, 1602 w, 1580 w, 1484 s, 1468 s, 1452 s, 1407 m, 1383 w, 1214 m, 1162 m, 1150 m, 1127 w, 1088 m, 1048 m, 1033 m, 967 s, 940 w, 827 s, 794 m, 770 s, 753 m, 695 m. Calc. for C_16_H_19_Cl_2_CrO: C, 54.87%; H, 5.47%. Found: C, 54.64%; H, 5.50%. HRMS Calc. for C_16_H_19_Cl_2_CrO = 349.0218. Found: C_16_H_19_Cl_2_CrO = 349.0215.

Synthesis of complex **3**: Ligand **3a** was prepared according to the literature [27]. To a solution of 1.28 g (5.04 mmol) ligand **3a** in 20 mL THF in the flask, a solution of butyl lithium 4.13 mL (1.22 mol/L, 5.04 mmol) in THF was dropwise added at −78 °C. After complete addition, the reaction mixture was stirred for 12 h. Then the resulting solution was added to the suspension of CrCl_3_ (THF)_3_ 1.87g (5.04 mmol) in 45 mL of THF at −78 °C. The mixture was stirred overnight. The color of the reaction mixture changed from purple to blue then to dark blue. After the solvent was removed under vacuum, toluene was added to precipitate LiCl, and the organic layer was concentrated to 30 mL. At low temperature, 0.32 g dark blue needle crystals were obtained in 16.9% yield. EI-MS (m/e): 375 (1, M), 340 (17, M-Cl), 291 (14, M-2Cl-CH_3_), 219 (4, M-CH_3_-2Cl-(CH_2_)_5_). IR (cm^−1^, KBr): 3092 m, 3107 m, 3029 w, 2970 m, 2920 s, 2855 m, 2829 w, 1637 s, 1480 s, 1460 w, 1435 w, 1407 m, 1368 m, 1137 m, 1074 m, 1040 s, 921 m, 827 s, 725 m, 651 m. Calc. for C_18_H_21_Cl_2_CrO: C, 57.46%; H, 5.63%. Found: C, 56.99%; H, 5.40%. HRMS Calc. for C_18_H_21_Cl_2_CrO = 375.0375. Found: C_18_H_21_Cl_2_CrO = 375.0394.

Synthesis of complex **4**: Ligand **4a** was prepared according to the literature [27]. To a solution of 0.93 g (4.30 mmol) ligand **4a** in 20 mL THF in the flask, a solution of butyl lithium 3.80 mL (1.22 mol/L, 4.30 mmol) in THF was dropwise added at −78 °C. After complete addition the reaction mixture was stirred for 12 h. Then the resulting solution was added to the suspension of CrCl_3_ (THF)_3_ 1.61 g (4.30 mmol) in 45 mL of THF at −78 °C. The mixture was stirred overnight. The color of the reaction mixture changed from purple to blue then to dark blue. After the solvent was removed under vacuum, toluene was added to precipitate LiCl, and the organic layer was concentrated to 30 mL. At low temperature, 0.36 g dark blue needle crystals were obtained in 27.1% yield. EI-MS (m/e): 309 (29, M), 273 (28, M-Cl), 244 (100, M-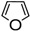), 239 (7, M-2Cl). IR (cm^−1^, KBr): 3112 m, 3096 m, 2970 s, 2931 m, 2878 m, 1613 w, 1502 m, 1468 m, 1414 m, 1381 w, 1261 m, 1159 s, 1105 m, 1049 m, 1014 s, 921 m, 838 s, 805 w, 724 s, 598 m. Calc. for C_13_H_15_Cl_2_CrO: C, 50.34%; H, 4.87%. Found: C, 50.21%; H, 5.03%.

Synthesis of complex **5**: Ligand **5a** was prepared according to the literature [27]. To a solution of 0.93 g (4.3 mmol) ligand **5a** in 20 mL THF in the flask, a solution of butyl lithium 3.80 mL (1.22 mol/L, 4.3 mmol) in THF was dropwise added at −78 °C. After complete addition the reaction mixture was stirred for 12 h. Then the resulting solution was added to the suspension of CrCl_3_ (THF)_3_ 1.61 g (4.3 mmol) in 45 mL of THF at −78 °C. The mixture was stirred overnight. The color of the reaction mixture changed from purple to blue then to dark blue. After the solvent was removed under vacuum, toluene was added to precipitate LiCl, and the organic layer was concentrated to 30 mL. At low temperature, 23.1 mg dark blue needle crystals were obtained in 16.1% yield. EI-MS (m/e): 335 (67, M), 299 (100, M-Cl), 261 (51, M-2Cl), 214 (34, M-2Cl-Cr). IR (cm^−1^, KBr): 3134 w, 3107 m, 2934 s, 2856 m, 1656 w, 1637 w, 1623 w, 1496 m, 1461 m, 1409 m, 1223 w, 1198 w, 1156 m, 1114 w, 1045 w, 1012 m, 936 w, 885 w, 830 s, 748 s, 598 w, 503 w, 423 w. Calc. for C_15_H_17_Cl_2_CrO: C, 53.59%; H, 5.10%. Found: C, 53.20%; H, 5.22%.

Synthesis of complex **6**: Ligand **6a** was prepared according to the literature [27]. To a solution of 0.93 g (4.3 mmol) ligand **6a** in 20 mL THF in the flask, a solution of butyl lithium 3.80 mL (1.22 mol/L, 4.3 mmol) in THF was dropwise added at −78 °C. After complete addition the reaction mixture was stirred for 12 h. Then the resulting solution was added to the suspension of CrCl_3_ (THF)_3_ 1.61 g (4.3 mmol) in 45 mL of THF at −78 °C. The mixture was stirred overnight. The color of the reaction mixture changed from purple to blue then to dark blue. After the solvent was removed under vacuum, toluene was added to precipitate LiCl, and the organic layer was concentrated to 30 mL. At low temperature, 23.1 mg dark blue needle crystals were obtained in 16.1% yield. EI-MS (m/e): 309 (13, M), 273 (100, M-Cl), 239 (21, M-2Cl), 187 (13, M-2Cl-Cr). IR (cm^−1^, KBr): 3104 s, 2975 s, 2935 m, 2875 m, 1608 m, 1552 m, 1477 m, 1447 w, 1409 m, 1242 m, 1217 m, 1108 s, 1043 m, 1023 m, 958 m, 835 s, 786 s, 679 w. Calc. for C_13_H_15_Cl_2_CrO: C, 50.34%; H, 4.87%; Found: C, 51.04%; H, 5.07%.

Synthesis of complex **7**: Ligand 7**a** was prepared according to the literature [29]. To a solution of 0.73 g (3.7 mmol) ligand **7a** in 20 mL THF in the flask, a solution of butyl lithium 2.9 mL (1.22 mol/L, 3.7 mmol) in THF was dropwise added at −78 °C. After complete addition the reaction mixture was stirred for 12 h. Then the resulting solution was added to the suspension of CrCl_3_ (THF)_3_ 1.39 g (3.70 mmol) in 45 mL of THF at −78 °C. The mixture was stirred overnight. The color of the reaction mixture changed from purple to blue then to dark blue. After the solvent was removed under vacuum, toluene was added to precipitate LiCl, and the organic layer was concentrated to 30 mL. At low temperature, 145 mg dark blue needle crystals were obtained in 12.6% yield. EI-MS (m/e): 335 (67, M), 299 (100, M-Cl), 261 (51, M-2Cl). IR (cm^−1^, KBr): 3107 m, 2934 s, 2856 m, 1656 w, 1637 w, 1623 w, 1496 m, 1461 m, 1409 m, 1223 w, 1198 w, 1156 m, 1114 w, 1074 w, 1046 w, 1012 m, 936 w, 885 w, 830 s, 748 s, 598 s, 503 s, 423 s. Calc. for C_12_H_13_Cl_2_CrS = 310.95: C, 46.17%; H, 4.20%. Found: C, 45.91%; H, 4.23%.

### 3.3. Polymerization Procedure

A 50 mL flask equipped with an ethylene inlet, magnetic stirrer, and vacuum line. The flask was filled with proper volume of freshly distilled solvent. MAO was added, and the flask was placed in a bath at the desired polymerization temperature for 10 min. The polymerization reaction was started by adding a solution of the catalyst precursor with a syringe. The polymerization was carried out for 30 min and then quenched with 3% HCl in ethanol (50 mL). The precipitated polymer was filtered and then dried overnight in a vacuum oven at 80 °C. ^1^H-NMR spectra were recorded on a Varian GRMINI-400 spectrometer (Fitchburg, MA, USA) in 1,2-dichlorobenzene at 100 °C. Molecular weight and molecular weight distribution (*M*_w_/*M*_n_) values were obtained from Waters-208 LC/GPC chromatograms (Milford, MA, USA) employing polystyrene standards for calibration. Analysis was carried out by using 1,2-dichlorobenzene at high temperature (140 °C).

## 4. Conclusions

A series of novel oxygen or sulfur functionalized cyclopentadienylchromium complexes were prepared and characterized. In the presence of MAO, these complexes show moderate to high activities in catalyzing ethylene polymerization and afford high molecular weight polymers. The electronic properties and steric hindrances of the substituents of the ligands remarkably affect the catalytic activities of these complexes, and complex **7** with the thiofuryl substituted cyclopentadienyl ring of the ligand exhibits the highest activity among them. The highest activity for ethylene polymerization was achieved as 1.96 × 10^6^ g PE/mol Cr·h by complex **7**. These results and the easy modification feature of these complexes are helpful in developing efficient catalysts which have potential industrial applications.

## Figures and Tables

**Table 1:** Polymerizations of ethylene using cyclopentadienylchromium complex **1**–**7** ^a^.

Entry	Cat. (amt of M (μmol))	Al/M	PE (mg)	Activity (10^5^ g/(mol-M⋅h))	*M*_η_^b^ (10^4^ g/mol)	*T*_m_/*T*_c_^c^ (°C)
1	Cp_2_TiCl_2_ (5.05)	1000	725	2.87	--	--
2	Cp_2_ZrCl_2_ (5.05)	1000	4090	16.2	--	--
3	**1** (5.05)	1000	66	0.39	8.96	133.4/109.7
4	**1** (5.05)	2000	326	1.29	5.62	134.6/110.5
5	**2** (5.05)	1000	169	0.67	7.73	133.7/110.2
6	**2** (5.05)	2000	804	3.19	4.40	132.5/109.1
7	**3** (5.05)	1000	30	0.12	--	--
8	**3** (5.05)	2000	40	0.16	--	--
9	**4** (5.05)	1000	286	1.16	15.8	135.2/110.8
10	**4** (5.05)	2000	1619	6.41	6.88	134.5/111.9
11	**5** (2.39)	1000	13	0.18	--	--
12	**5** (2.39)	2000	20	0.11	--	--
13	**6** (2.10)	1000	153	1.46	10.8	133.2/109.5
14	**6** (2.10)	2000	544	5.18	6.74	133.6/109.2
15	**7** (2.89)	1000	1593	11.0	14.0	134.2/110.7
16	**7** (2.89)	2000	2834	19.6	8.32 ^d^	134.7/111.3

^a^ Conditions: toluene as solvent, *V*_total_ = 50 mL, Temp. = 25 °C, MAO as cocatalyst, 0.2 MPa of ethylene, 30 min; ^b^ Intrinsic viscosity was determined in decahydronaphthalene at 135 °C by Ubbelohde viscometer technique, and the viscosity average molecular weights were calculated using the relation [33]: [η] = 6.67 × 10^−4^*M*_η_^0.67^, in unit of 10^4^ g/mol; ^c^ Determined by DSC at a heating rate of 10 °C min^−1^; ^d^ *M*_w_ and *M*_w_/*M*_n_ were determined by GPC, using 1,2-dichlorobenzene as solvent at 140 °C, in unit of 10^4^ g/mol. For entry 16, *M*_w_ = 4.92 ×10^4^ g/mol, *M*_w_/*M*_n_ = 1.89.

## Supplementary Materials

Supplementary Materials are available online. The figure of the infrared spectra of comparison of complex **1** and ligand **1a**; the ^1^H-NMR spectrum of typical polymer obtained with **7**/MAO (entry 16).
