# Supplementary material for: Synthesis and Characterization of Constrained Geometry Oxygen and Sulphur Functionalized Cyclopentadienylchromium Complexes and Their Use in Catalysis for Olefin Polymerization
Source: Molecules. 2017 May 22;22(5):856. doi: 10.3390/molecules22050856 (PMC6154690; doi:10.3390/molecules22050856)
Supplement: Supplementary file 1 [file molecules-22-00856-s001.pdf]

Supporting information for

Synthesis and Characterization of Constrained Geometry Oxygen and Sulphur Functionalized Cyclopentadienylchromium Complexes and Their Use in Catalysis for Olefin Polymerization

Ruiguo Zhao[1, 2], Jun Ma[2], Hao Zhang[1, 2, *], Jiling Huang[2, *]

1 School of Chemistry and Chemical Engineering, Inner Mongolia University, Hohhot 010021, P.R. China; zhaoruig@imu.edu.cn (R.G. Z); haozhang@imu.edu.cn (H. Z.)

2 The Laboratory of Organometallic Chemistry, East China University of Science and Technology, Shanghai 200237, P.R. China.; jlhuang@ecust.edu.cn

* Correspondence: haozhang@imu.edu.cn (H. Z.); jlhuang@ecust.edu.cn (J.L. H.)

Tel.: +86-0471-4994406 (H. Z.); +86-021-64253519 (J.L. H.)

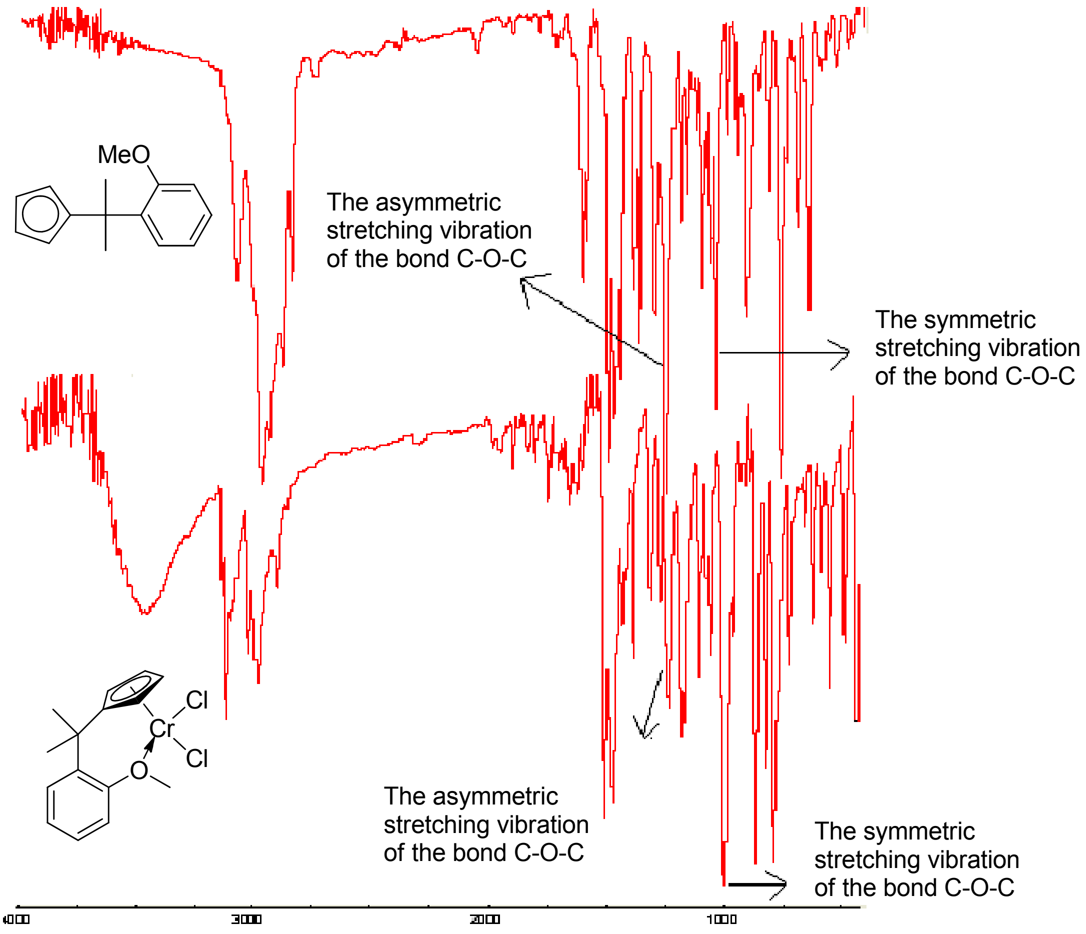

Figure S1. IR spectra of comparison of complex **1** and ligand 1a.

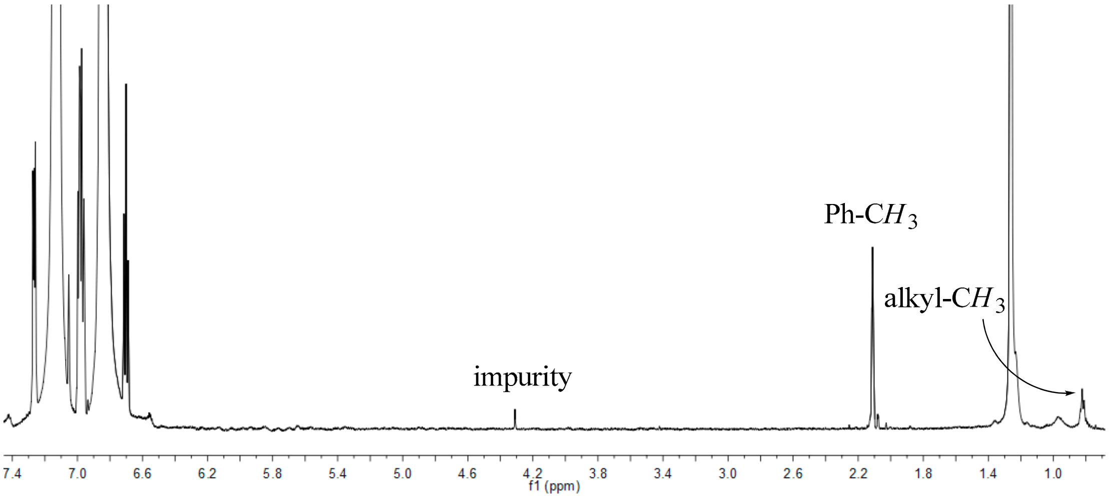

Figeur S2. ^{1}HNMR spectrum of polymer obatined by complex **7** (entry 14, 400 MHz, *o*-$C_6H_4Cl_2$: C_6D_6 = 4 : 1, 100 °C).
